# What Is Nutritious Snack Food? A Comparison of Expert and Layperson Assessments

**DOI:** 10.3390/nu9080874

**Published:** 2017-08-14

**Authors:** Tamara Bucher, Christina Hartmann, Megan E. Rollo, Clare E. Collins

**Affiliations:** 1 Institute of Food, Nutrition and Health (IFNH), ETH Zürich, Universitätstrasse 22, Zürich 8092, Switzerland; christina.hartmann@hest.ethz.ch; 2 School of Health Sciences, Faculty of Health and Medicine, University of Newcastle, Callaghan, NSW 2308, Australia; Megan.rollo@newcastle.edu.au (M.E.R.); clare.collins@newcastle.edu.au (C.E.C.); 3 Priority Research Centre in Physical Activity and Nutrition, University of Newcastle, Callaghan, NSW 2308, Australia

**Keywords:** health perception, portion size, food labels, healthy choice, language analysis, mixed-method design, health claim

## Abstract

The term “nutritious” is being increasingly used by product manufacturers but the term is not currently regulated as a nutrition claim. It is unclear how lay consumers and experts define and interpret the term or how they evaluate the “nutritiousness” of various foods. To address this evidence gap, a mixed methods design was applied and both nutrition experts (*n* = 206) and lay participants (*n* = 269) provided definitions of the term “nutritious” and evaluated the “nutritiousness” of 20 different snack foods in a cross-sectional survey. Definitions were analysed using Leximancer and snack evaluations were compared both between groups and with nutrient profile scores (UK Ofcom and Australian Health Star Rating). Expert and lay definitions differed considerably, with experts using terms such as nutrient-density, macro- and micronutrients, kilojoules/Calories, while lay consumers used descriptions such as fuel, fresh, natural, body needs, and functioning. Snack evaluations were highly correlated between groups (*R*_s_ > 0.89, *p* < 0.001) and between nutrient profile scores (*R*_s_ > 0.75, *p* < 0.001). However, mean perceptions significantly differed for 18 out of 20 foods with the largest difference for yoghurts (*p* < 0.05). There are discrepancies between expert and lay perceptions of snack foods and the definition of the term “nutritious”. The results highlight the need for an agreed definition and the potential regulation of the term “nutritious” in food marketing.

## 1. Introduction

Choosing a variety of healthy foods and adequate portion sizes is important for weight management and overall health [1,2,3]. A systematic review of 28 studies, which examined the relationship between diet quality, morbidity, and mortality, demonstrated that higher diet quality was consistently inversely related to all-cause mortality [1]. In addition to choosing a variety of foods to optimise intake of essential nutrients, selecting adequate portion sizes is important in terms of energy intake. However, research shows that people tend to focus more on the type of food consumed rather than the amount [4,5]. To help consumers choose nutritious foods in healthy proportions, we need to understand how consumers perceive and evaluate food and how they define different terms, such as “healthiness” and “nutritiousness”. Knowing how people interpret and implement nutrition information is important for stakeholders who aim to promote health by communicating nutrition information on how to improve food selection and portion size choices.

Informing consumers via nutrition labels and health claims is one possible avenue to promote population health. Consumer perceptions and responses to health claims have been studied extensively [6,7,8], and health and content claims are strictly regulated within the European Union, Australia, and other countries. However, more general terms such as “healthy”, “natural”, “nutritious” and images indicating healthy options or health benefits [9] are poorly regulated yet frequently used for marketing purposes. In the USA, the US Food and Drug Administration (FDA) regulates the use of the term “healthy” [10] and this encourages manufacturers to advertise with non-legislated terms such as “wholesome” or “nutritious” [11].

To date, the term “nutritious” or “nutritious food” lacks a clear definition [12] and it is unknown how consumers interpret it. A “nutritious” claim may mislead consumers into thinking that a product is good for their health, even if it contains unfavourable amounts of sugar, fat, or sodium. This has previously been observed with claims such as “low fat” or “high in vitamin C” [13,14,15].

To objectively evaluate the “nutritiousness” of different foods and meals, different approaches to quantify and score products based on nutrient density have been suggested [16]. Nutrient profile scores, such as the Ofcom nutrient profile score [17] and the Australian Health Star Rating [18], use a formula to account for “healthy” and “unhealthy” food components. The term “nutritious” could therefore be defined as, “to contain a high density of preferable nutrients”, i.e., nutritious food should provide a high percentage of essential nutrients relative to the energy content. Expert opinion has been used in the development and validation process of nutrient profile scores [19,20,21,22]. However, commonly there are discrepancies between what experts and lay consumers consider to be relevant nutrition information on food labels [23].

Several studies have investigated the criteria that different consumer groups use when determining the “healthiness” of various foods [24,25] and beverages [26]. It has been found that sugar, fruit/nut content, fat, and fibre (for young adults and women) are significant predictors. A recent study in adolescents found that sugar, fruit, nut, and total fat content were important predictors for determining the healthiness of snack foods [27]. However, how lay consumers perceive nutrition and portion size compared to experts is less clear.

In the current study, we investigate the term “nutritiousness” rather than healthiness, as this is an expression increasingly used for marketing purposes. To date, it is unclear how consumers interpret the term “nutritious” and which criteria they use to determine whether a food is nutritious or not. In order to promote healthy eating and to design nutrition information panels that are relevant to consumer needs, it is important for nutrition experts and policy makers to understand how the general population define and interpret the term “nutritious”. Therefore, the aim of the current study was to explore how nutrition experts and lay consumers define the term “nutritious” and how they evaluate the “nutritiousness” of various snack foods. In addition, we examined the role of portion size (amount of food) and its relationship to the term “nutritious”. The focus of this study was on the evaluation of snack foods as snacking has become an increasingly common eating habit [28] which contributes to a high proportion of overall energy [29] and can therefore significantly impact dietary quality. Furthermore, snack foods often come as packaged convenience products that carry health and nutrition claims and the consumed portion size is frequently determined by the unit size (e.g., one muesli bar).

## 2. Material and Methods

All subjects gave their informed consent for inclusion before they participated in the study. The study was conducted in accordance with the Declaration of Helsinki, and the protocol was approved by the Ethics Committee of the University of Newcastle (H-2015-0228).

### 2.1. Snack Selection

A team of nutritionists and dietitians selected 20 snacks commonly consumed in Australia. The snacks were selected to (1) represent foods commonly consumed as snacks in Australia [30,31]; (2) represent foods from all food groups; and (3) vary in their nutrient profile score [17].

The characteristics of the snacks included in the study (nutrient information per 100 g, nutrient profile scores and portion sizes) can be found in Table A1. Two portion sizes (small 50% of large size) that could be consumed within one sitting were chosen. For the survey, photos of all snack portions (*n* = 40) were taken.

### 2.2. Survey and Data Collection

Two separate surveys were used to collect data from nutrition experts and lay participants. Participants of both surveys could enter a draw to win one of two vouchers from local retail shops.

### 2.3. Expert Participant Survey

In Australia, Accredited Practicing Dietitians (APDs) are considered the experts in providing nutrition and dietary advice and medical nutrition therapy [32]. The APD credential requires a university qualification in an approved degree studying human nutrition and dietetics and membership with the governing body, the Dietitians Association of Australia (DAA). To maintain APD status, ongoing training, education, and professional development are required to ensure APDs are providing up-to-date and credible nutrition information [32].

**Figure A1:**
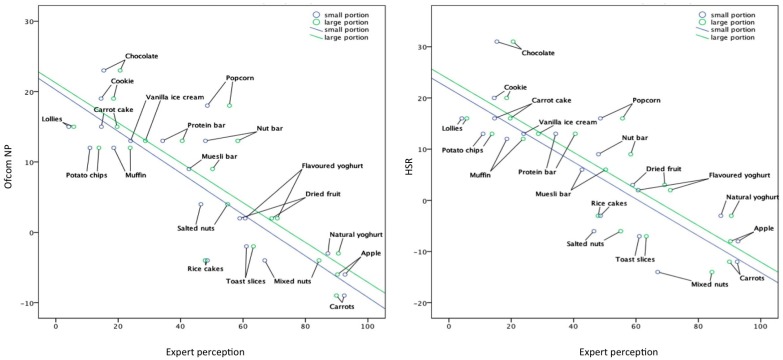
Perception of “nutritiousness” of 20 different snacks by nutrition experts (*N* = 206) versus Ofcom Nutrient Profile scores (Ofcom NP; **left**) and Health Star Rating score (HSR, **right**). Experts evaluated 20 snacks in a small portion (blue) size and a large (green) portion size on a scale ranging from (not nutritious) to 100 (very nutritious). Lines indicate linear regressions. Biases for certain foods are visible such as chocolate, which is rated as more nutritious than expected from nutrient profile scores and “lollies” were ranked as less nutritious.

The nutrition experts for this study were recruited via a survey link that was sent within an email newsletter of the Dietetic Association of Australia (DAA). Expert participants were asked to provide a definition of the term “nutritious” within an open-ended question. Subsequently, they were asked to rate the nutritiousness of 20 different snack foods in two portion sizes on a scale ranging from 0 (not nutritious) to 100 (very nutritious). The big snack portion was double the size of the small portion. A nutrition information panel with the amounts of energy (kJ and kcal), protein (g), fat (g), saturated fat (g), sugar (g), dietary fibre (g), and sodium (mg) per portion were indicated next to a picture of the snack. An example of one snack presented in two portion sizes can be found in Figure A1. Portion sizes, nutrient content, and nutrient profile scores of all snacks are reported in Table A1.

Participants were advised that the food portions would be consumed in one sitting as a snack in between meals by a 30 year-old woman who was moderately active and a normal-weight. No further information was provided on the overall diet of this individual apart from the snack. They were also instructed that they could use the “forward” and “back” buttons in the survey to change their ratings until they had completed their evaluation of all the food portions. After the snack rating, participants’ practical nutrition knowledge about balanced eating (PKB-7) [33] and the energy content of meals (PKEM-11) [34] as well as information about their professional education were assessed.

### 2.4. Lay Participant Survey

Lay participants were recruited via advertisements on social media in July and August 2016. The first part of the survey was identical to the expert survey. Lay participants were asked to define the term “nutritiousness”, to rate the nutritiousness of the 20 snack foods in two different portion sizes, and to complete the practical knowledge questions.

### 2.5. Nutrient Profile Models

We compared the perceived nutritiousness to the Ofcom Nutrient Profiling (NP) model [17] and the Health Star Rating (HSR) as objective comparators [18]. The Ofcom NP model sums negative points for nutrients that should be avoided (sugar, sodium, saturated fat, total energy) and subtracts points for positive nutrients (fruits, vegetables and nuts, fibre, protein) [17].

The profiling system went through several stages of development and the performance of various calculation methods was evaluated through a comparison of the scores with the opinions of an expert panel for the 120 food products [19,20,35]. A lower score indicates a more nutritious food product. The Ofcom NP model was used to regulate the advertisement for unhealthy food products to children in the UK. The cut-off to deem a food as less healthy was set at >4. For further information on methodology, see Rayner et al. [17]. The Ofcom NP model also served as a basis for the development of the Australian Health Star Rating (HSR) system by the Food Standards Australia New Zealand (FSANZ). The HSR uses the same underlying formula as the Ofcom NP but with different and extended cut-offs. Furthermore, food category specific cut-offs were introduced by the HSR. The HSR system is used to calculate the number of Health Stars, which can be displayed as a front of pack label on a voluntary basis by food manufacturers [18]. The Ofcom NP scores and the HSR scores of all snacks are reported in Table A1.

### 2.6. Data Analysis

The “nutritious” definitions were analysed with the language analysis software Leximancer (version 4; Leximancer Pty Ltd., Brisbane, Australia). Leximancer is a text analytics tool that can be used to analyse the content of text to display the extracted information visually. It can quantify and display the conceptual structure of the key concepts, as well as the frequency of co-occurrence of key concepts (or main relationships between key concepts). The software uses an iterative numerical model that operates to render large amounts of language data into a complex network system. In other words, it automatically selects a rank list of key words in a set of documents on the basis of their frequency and co-occurrence. The program then builds a “thesaurus”, which extends the seed words into weighted terms called “concepts”. The text is classified into text blocks to produce a concept index and a concept co-occurrence matrix. The software then calculates the relative co-occurrence frequencies of the concepts to obtain an asymmetric co-occurrence matrix. The matrix is used to produce a concept map informed by a clustering algorithm [36].

Lay participants and experts rated the nutritiousness of 20 snacks in two portion sizes on a scale ranging from 0 (not nutritious) to 100 (very nutritious). Mean values of experts and lay participants were compared using independent sample *t*-tests. Differences between portions sizes within groups were compared using dependent sample *t*-tests. Bonferroni corrections were applied to account for multiple testing for mean perceptions of different snack portion sizes and mean snack perceptions between the two study groups for all foods. Correlations between perceived nutritiousness (small and large portion, lay and expert evaluation) and the Ofcom Nutrient Profiling (NP) scores [17] and the Health Star Rating (HSR) [18] were investigated.

Statistical analysis was conducted using IBM SPSS Statistics, Version 22 (SPSS. Inc., Chicago, IL, USA). For normally distributed data, means (*M*), standard deviations (*SD*), and Pearson correlation coefficients (*R*) are reported. For non-parametric data, the median and Spearman correlation coefficients (*R_S_*) were reported and Wilcoxon signed ranked tests were used for comparisons of dependent data within groups. *T*-tests (T_(df)_) were used for comparison of independent data. All tests were based on a 0.05 significance level.

## 3. Results

### 3.1. Participant Characteristics

The data set was cleaned in alignment with recommendations [37] with incomplete responses, duplicates and those where time to complete the survey was less than 50% of the median duration. The final sample contained 476 respondents, 207 experts (97%, *n* = 201 female) and 269 lay participants (77%, *n* = 207 female). Thirty-nine percent (*n* = 80) belonged to the New South Wales DAA branch, 24% (*n* = 49) to the Victoria branch, 15% (*n* = 31) to the Queensland branch, and the remainder (22%, *n* = 47) belonged to other DAA branches. Ninety-six percent (*n* = 197) were currently working with 67% working full time and 28% working part time. Thirty-two percent (*n* = 65) graduated more than 10 years ago, 21% (*n* = 43) between 6 and 10 years, 18% (*n* = 38) between 3–5 years and 29% (*n* = 60) within the last three years. In the lay participant group, 70% (*n* = 188) had higher (University) education and 93% (*n* = 249) were Australian citizens. Applied nutrition knowledge on balanced eating (M_expert_ = 4.7, SD = 0.9, M_lays_ = 3.9, SD = 1.2, T_(473)_ = 14.6, *p* < 0.001) and meal energy content (M_expert_ = 8.1, SD = 1.2, M_lays_ = 6.2, SD = 1.7, T_(473)_ = 13.5, *p* < 0.001) was significantly higher in the experts compared to the lay participants. Both groups had a similar mean age; the experts’ mean age was 35.6 (SD = 11.0) years and the lay participants mean age was 34.7 (SD = 14.0) years. The lay participants had a mean BMI of 24.7 (SD = 4.9) kg/m^2^. BMI was not assessed in the expert group.

### 3.2. Definition of “Nutritious”

**Figure 1:**
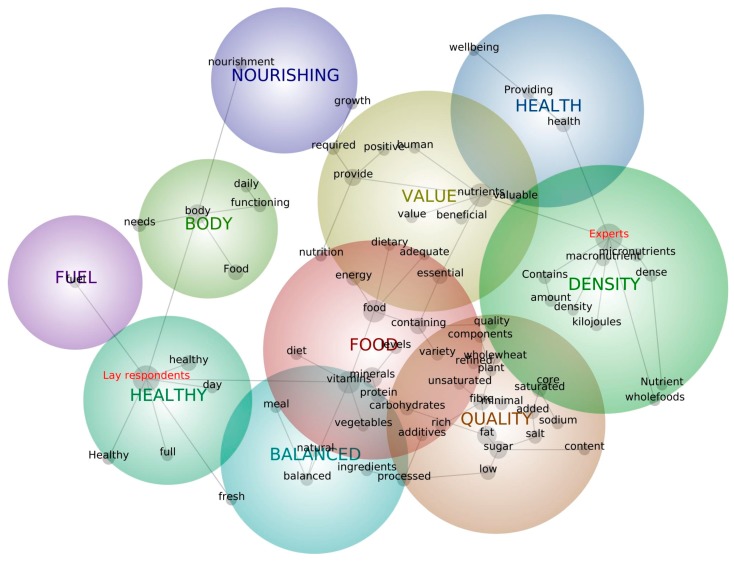
Concepts and identified themes related to the term “nutritious”. “Nutritious” definitions of lay participants and experts were analysed using the topical (linear) clustering algorithm in Leximancer. The lines between the concepts (grey circles) show typical pathways between the concept terms (black print) in the language data. The size of the grey circles indicates the overall relative frequency of concepts. The closeness of concepts indicates their context. Note that some concepts appear several times on the map depending on their context (e.g., “healthy” is used in context with “body” and with “food”). Nine themes were identified (coloured circles).

The participants’ understanding and perception of the term “nutritious” was investigated using an open-ended question. Figure 1 provides a map of the key concept words using the topical (linear) clustering algorithm in Leximancer. Nine main themes were identified in the definitions with the themes “fuel”, “healthy”, “body”, “nourishing”, and “balanced” appearing more often in lay definitions and “health”, “nutrient”, “density”, “quality”, and “value” appearing more frequently in expert definitions. The concepts in the more general theme of “food” appeared in both definitions. The expert and lay definitions differed considerably, with experts frequently using terms such as nutrient density, macro- and micronutrients, kilojoules/calories, and unsaturated fat and lay participants using holistic descriptions such as fuel, fresh, natural, body needs and functioning. The most frequently mentioned words in the expert definitions were “nutrient” (*n* = 115, 55.6%), “provide” (*n* = 73, 35.3%), and “fat” (*n* = 49, 23.7%); whilst “body” (*n* = 100, 37%), “healthy” (*n* = 96, 35.6%) and “nutrient” (*n* = 73, 27%) were the most frequently mentioned words in the lay definitions (Table 1).

### 3.3. Evaluation of the Nutritiousness of Snacks

**Figure 2:**
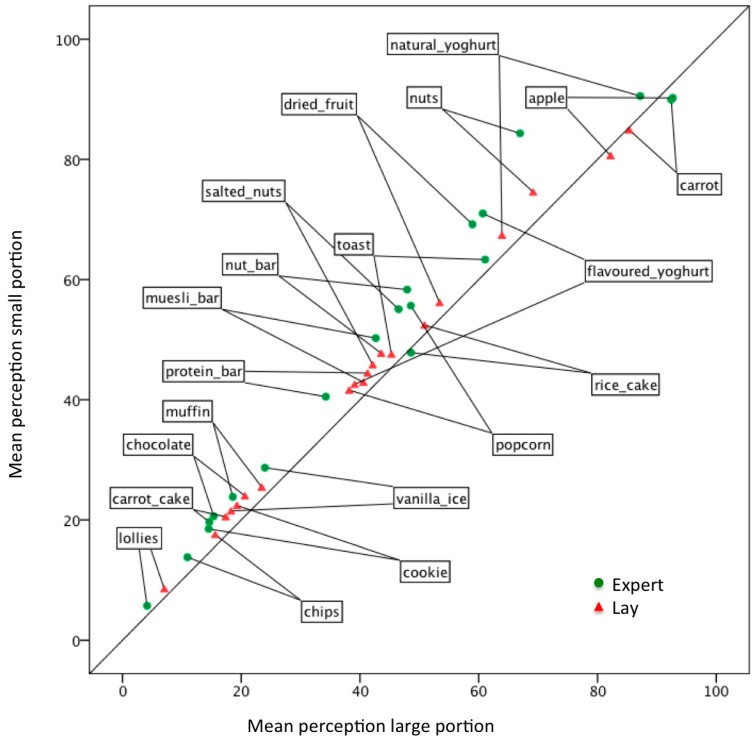
Perception of “nutritiousness” of different snacks (*N* = 20) by nutrition experts (*N* = 206) and lay participants (*N* = 269). Large differences were found for lay and expert evaluations of yoghurt and toast, smaller differences were found for discretionary foods. Note that for most foods, a smaller portion was evaluated as more nutritious (above the reference line).

The evaluations of the snacks were highly correlated between experts and lay participants (*R*_S_ > 0.89, *p* < 0.001). Additionally, both were highly correlated with the two nutrient profile scores (*R*_S_ > 0.82, *p* < 0.001) (Table 2). However, biases were found for certain foods such as “chocolate”, which was rated as more nutritious than expected from nutrient profile scores and “lollies”, which was ranked as less nutritious compared to the nutrient profile scores (Plots of the HSR and Ofcom nutrient profile scores versus expert evaluations can be found in Figure A1). The mean “nutritiousness” evaluations differed significantly between experts and lay participants for 18 out of 20 foods. The largest differences were found for natural and flavoured yoghurts and toast, with experts evaluating these foods as more nutritious (Table 3 and Figure 2). Differences were smaller for the discretionary foods such as lollies, carrot cake, and rice cakes. Overall, both the experts and lay participants evaluated carrots and apples as the most nutritious foods and lollies as the least nutritious. Compared with the lay participants, the experts evaluated snack foods with better nutrition profile scores as more nutritious and foods with a lower nutrient profile score as less nutritious (Table 3).

Different sized portions of the same snacks were evaluated differently for all foods except rice cakes by the experts and rice cakes, carrots, apples, and lollies by the lay participants (Table 3). For most foods, except carrots and apples, both experts and lay participants rated a smaller portion as more nutritious. Differences between large and small portions were biggest for the mixed nuts. On average, the experts perceived bigger differences in nutritiousness between large and small portions compared with lay participants (M_expert_ = −5.2, SD = 4.7, M_lay_ = −2.4, SD = 1.6, T_(19)_ = 3.6, *p* < 0.005) (Table 3).

## 4. Discussion

Definitions of “nutritious” provided by expert and lay participants differed. In line with how nutrient profile scores are defined, experts used terms such as micro- and macronutrients, vitamins and minerals, as well as nutrient density and concentration. Lay participants, however, used more holistic and descriptive terms such as body needs, fuel, and fresh. Nutrient profiling systems and labels based on these systems are now implemented in several countries. However, the term “nutritious” is not currently regulated in most countries and little is known about how consumers interpret the term. Differences in the perception of what constitutes a healthy product and the description of the term “nutritious” indicates that an agreement on the term could be useful for consumers and for the potential regulation of the claim in product marketing. These findings are in accordance with a recent study with young adults by de Vlieger et al., which found that young adults describe a nutritious food as one that is “healthy” or “low in sugars, but high in vitamins and minerals”, or should “provide your body with what it needs” [38]. We suggest that in accordance with established nutrient profile scores, which correlate highly between lay and expert opinions in several countries, the term “nutritious” should be defined in relation to nutrient density.

Experts and lay consumers often disagree about what nutrition information is relevant [23]. Nevertheless, the current findings show that Australian nutrition experts and lay consumers generally agree on the nutritiousness of several snack foods and more importantly, their perceptions are highly correlated with nutrition profile scores, which consider total energy content, saturated fat, sodium, protein, fibre, and fruit/vegetable/seed and nut content. This is in line with previous studies conducted with adults and adolescents in Switzerland, which found high correlations between the Ofcom nutrient profile score and consumers perceptions [25,27]. However, there is a bias for certain foods, which should be further explored.

The Ofcom nutrient profile score was developed by testing different formulas for their ability to predict scores from nutrition experts. The best algorithm was then tested and further refined using evaluations of >700 nutrition and dietetic professionals in the UK [17,19,20]. The Ofcom NP model was used to regulate the advertisement of unhealthy food products to children in the UK. The Ofcom NP model also served as a basis for the development of the Australian Health Star Rating (HSR) system by Food Standards Australia New Zealand (FSANZ) [18]. The HSR is used by FSANZ to calculate the number of health stars for the Health Star Label. The Australian Health Star Label is a front of pack label, which was developed to provide consumers with easy to understand information about the healthiness of food and beverage products and to allow a quick comparison of products at the point of purchase. The label is voluntary and should serve as an incentive for food manufactures to reformulate their products to score a higher Health Star Rating [18]. The HSR calculation system uses the same underlying formula as the Ofcom NP, but the cut-offs were modified. These modifications, as well as different cultural backgrounds of the nutrition expert panel, could have led to a variation in agreement. However, the data in this study indicated that correlations of experts’ product ratings with both nutrient profile scores were high and comparable to Ofcom nutrient profile score (Table 2).

There were some considerable differences in the perception of the nutritiousness of yoghurts between experts and lay participants. This indicated that consumers may be confused about the nutritional value of different yoghurts and this is an area where communication of nutrition content could be improved. Health Star Labels for this product category might be useful for providing such consumer guidance.

Nutrient profile scores currently do not consider portion sizes, instead they are based on the nutrient content per 100 g of the food product. In this current study, participants evaluated the nutritiousness of both small and large portions of 20 different snack foods and evaluations from the experts and lay participants differed for most portion sizes, indicating that both groups considered portion size in their evaluations. This finding suggests that portion size specific adjustments to the current nutrient profile score calculation system might be worth investigating. However, this may be less relevant for foods with a low energy density, such as rice cakes. Whilst smaller portions were generally considered to be more nutritious, the relationship is non-linear as larger portions of very nutritious foods such as carrots and apples were considered to be more nutritious. The current study was limited to a relatively small range of snack foods and therefore future testing of a larger variety of foods and portion sizes would be required to model any effect of portion size on perception. Future research should also evaluate whether portion size could be incorporated within the established nutrient profile scoring systems.

The current study has several limitations that need to be acknowledged. Firstly, for practical reasons the range of foods evaluated was limited to snack foods in amounts that could be consumed within one sitting and therefore this limited the variation in portion size. Secondly, both study groups were predominantly female and women are known to have an increased interest in health-related behaviours. Furthermore, the lay participant sample was not representative of the general Australian population and potentially, due to self-selection bias, people who participated in the study had a greater interest in nutrition. Despite this, there was a significant difference in applied nutrition knowledge between the expert and lay participants and the mean values were comparable to mean values obtained from a representative sample in previous studies [33,34].

To the best of our knowledge, the current study is the first to explore lay consumers perceptions of the term “nutritious” and is the first study to compare Australian experts and lay consumers’ evaluations of a variety of snack products with nutrient profile scores.

Future research could develop and test a portion-size adjusted nutrient profile score and investigate whether such food labels could guide portion size and portion control. This type of portion size adjusted nutrient profile score could be informed by standard serve sizes such as those used in Australia or by commonly consumed portions, such as those informed by national consumption data. However, portion size adjusted labels that are based on nutrient profiles need to be based on standardised volume measures with consistent units and terminologies to avoid consumer confusion [39].

## 5. Conclusions

The findings of the current study provide insight into consumer perceptions and may help to inform the design of more effective nutrition education materials and food labels to guide healthy choices. The results also highlight the potential need for definitions and regulation of the term “nutritious” in food marketing.

## Figures and Tables

**Table 1:** Word frequencies in lay and expert definitions.

	Expert (*n* = 206)			Lay (*n* = 269)		
	Word	Frequency (*n*)	%	Word	Frequency (*n*)	%
1	nutrient	115	55.6	body	100	37.0
2	provide	73	35.3	healthy	96	35.6
3	fat	49	23.7	nutrient	73	27.0
4	contain	49	23.7	vitamin	73	27.0
5	health	48	23.2	good	62	23.0
6	vitamin	46	22.2	mineral	53	19.6
7	mineral	43	20.8	provide	43	15.9
8	micronutrient	38	18.4	fat	36	13.3
9	fibre	38	18.4	contain	36	13.3
10	good	36	17.4	sugar	29	10.7
11	sugar	36	17.4	energy	25	9.3
12	body	35	16.9	full	23	8.5
13	macronutrient	34	16.4	protein	23	8.5
14	essential	29	14.0	health	22	8.1
15	sodium/salt	29	14.0	need	22	8.1
16	energy	27	13.0	essential	21	7.8
17	healthy	26	12.6	processed	19	7.0
18	dense	25	12.1	function	18	6.7
19	saturated	23	11.1	fibre	17	6.3
20	added	22	10.6	fresh	15	5.6
21	kilojoule	17	8.2	ingredient	14	5.2
22	amount	15	7.2	beneficial	14	5.2
23	protein	15	7.2	require	14	5.2
24	beneficial	14	6.8	diet	13	4.8
25	food group	13	6.3	fuel	13	4.8

**Table 2:** Spearman correlations between expert and lay evaluations and Ofcom nutrient profile (Ofcom NP) score and Health Star Rating (HSR) scores. Experts and lay participants evaluated 20 snacks in two portion sizes (small and large) on a scale ranging from 0 (not nutritious) to 100 (very nutritious). The Ofcom NP ranged from −9 (carrots) to 23 (chocolate). The HSR ranged from −12 (carrots) to 31 (chocolate). A cut-off for less healthy food was previously set at >4 for the Ofcom NP score [17].

		HSR Score	Ofcom NP	Expert Evaluation		Lay Evaluation	
				Small Portion	Large Portion	Small Portion	Large Portion
HSR score		1	0.955 **	−0.847 **	−0.805 **	−0.854 **	−0.843 **
Ofcom NP		0.955 **	1	−0.824 **	−0.759 **	−0.824 **	−0.812 **
Expert evaluation	small portion	−0.847 **	−0.824 **	1	0.974 **	0.923 **	0.916 **
	large portion	−0.805 **	−0.759 **	0.974 **	1	0.893 **	0.892 **
Lay evaluation	small portion	−0.854 **	−0.824 **	0.923 **	0.893 **	1	0.998 **
	large portion	−0.843 **	−0.812 **	0.916 **	0.892 **	0.998 **	1

**Notes:** ** Spearman correlation coefficients significant at *p* < 0.001.

**Table 3:** Experts (*N* = 206) and lay participants (*N* = 269) mean nutritiousness evaluations on a scale from 0 (not nutritious) to 100 (very nutritious), Ofcom nutrient profile (NP) scores and Health Star Rating (HSR) scores of *N* = 20 foods.

Snack	Portion Size (g)	Expert Perception		Lay Participant Perception		Difference between	HSR	Ofcom NP
		Mean	SD	Mean	SD	Evaluations		
Carrots	160	**92.0**	14.5	85.0	16.3	7.0 *	−12	−9
	80	**89.4**	17.4	84.3	18.3	5.1 *		
Apple	180	**92.2**	11.4	81.6	15.3	10.6 *	−8	−6
	90	**89.8**	13.8	80.0	17.1	9.7 *		
Rice cakes	50	48.3	22.2	50.6	22.0	−2.3	−3	−4
	25	47.4	23.2	51.8	24.2	−4.5		
Mixed nuts	80	**67.3**	21.4	**69.0**	19.1	−1.7	−14	−4
	40	**84.3**	14.7	**74.5**	16.9	9.8 *		
Natural yoghurt	240	**86.9**	13.4	**63.6**	20.7	23.3 *	−3	−3
	120	**90.1**	10.6	**67.0**	20.4	23.0 *		
Toast slices	50	**60.2**	20.0	**45.8**	20.4	14.4 *	−7	−2
	25	**62.4**	20.5	**47.8**	22.1	14.6 *		
Dried fruit	60	**59.5**	19.4	**54.8**	22.6	4.8	3	2
	30	**69.4**	18.8	**57.0**	22.4	12.5 *		
Flavoured yoghurt	240	**60.3**	19.2	**40.3**	20.7	20.0 *	2	2
	120	**70.6**	17.6	**43.7**	20.4	26.9 *		
Salted nuts	50	**46.9**	19.5	**43.0**	20.6	3.9	−6	4
	30	**55.3**	19.9	**46.6**	20.7	8.7 *		
Muesli bar	50	**43.2**	19.5	**41.8**	20.4	1.4	6	9
	25	**50.7**	20.7	**44.0**	20.4	6.7 *		
Muffin	180	**18.7**	15.1	**24.1**	18.0	−5.4 *	12	12
	90	**23.9**	16.6	**25.9**	17.7	−1.9		
Potato chips	50	**11.1**	13.5	**16.3**	15.3	−5.2 *	13	12
	25	**14.1**	14.0	**18.1**	16.4	−4.0		
Nut bar	60	**48.5**	20.3	**44.8**	20.6	3.6	9	13
	30	**58.7**	20.4	**48.7**	20.0	10.0 *		
Protein bar	60	**34.5**	21.1	**42.2**	20.6	−7.7 *	13	13
	30	**40.5**	23.1	**45.3**	21.1	−4.8		
Vanilla ice cream	80	**24.1**	18.9	**19.1**	15.7	5.1 *	13	13
	40	**28.9**	20.7	**22.3**	17.4	6.6 *		
Lollies	100	**4.3**	7.8	7.5	11.4	−3.2 *	16	15
	50	**6.1**	10.3	8.5	12.1	−2.4		
Carrot cake	200	**14.7**	14.8	**17.4**	16.4	−2.8	16	15
	100	**19.7**	15.5	**20.5**	15.5	−0.8		
Popcorn	40	**49.0**	20.0	**38.6**	20.9	10.3 *	16	18
	20	**55.6**	21.3	**42.0**	23.0	13.6 *		
Cookie	50	**14.9**	13.4	**19.9**	14.8	−5.0 *	20	19
	25	**18.8**	15.8	**22.9**	16.9	−4.1		
Chocolate	40	**15.7**	14.6	**21.6**	17.8	−5.9 *	31	23
	20	**21.1**	17.8	**24.7**	18.4	−3.6		

**Notes:** * Portion evaluated significantly different by experts and lay people (Independent sample *t*-tests with Bonferroni correction for 40 comparisons). Bold print: mean perception significantly differed between large and small portion (Dependent sample *t*-test with Bonferroni correction for 40 comparisons).

## Appendix A

**Table A1:** Characteristics of the snacks (*N* = 20) evaluated by experts and lay participants for the sorting task. Ofcom Nutrient Profile Score [17] Health Star Rating (HSR) scores and Health Stars were calculated [18] according to the nutrition information per 100 g derived from the AU food database (AUSNUT).

Snack	AUSNUT Food ID	Small Portion (g)	Large Portion (g)	Energy (kJ/100 g)	Saturated Fat (g/100 g)	Total Sugar (g/100 g)	Sodium (mg/100 g)	Fruit, Vegetable and Nuts (%)	Fiber (g/100 g)	Protein (g/100 g)	HSR	Health Stars	Ofcom NP Score	Ofcom Category
Carrot	13A11671	80	160	133	0	5	38	100	4	0.8	−12	★★★★★	−9	healthy
Apple	06D10559	90	180	239	0	11.9	1	100	2.3	0.3	−8	★★★★✧	−6	healthy
Rice cake	02C10116	25	50	1683	0.62	0.3	118	0	4.4	8.6	−3	★★★★	−4	healthy
Mixed nuts	11B10240	40	80	2642	4.03	3.9	4	100	7.6	19	−14	★★★★★	−4	healthy
Natural yoghurt	09C20042	120	240	241	0.19	5.9	84	0	0	6.6	−3	★★★★★	−3	healthy
Toast slice	02B10754	25	50	1247	0.79	2.3	520	0	7.4	13.2	−7	★★★★✧	−2	healthy
Dried fruit	06E10091	30	60	1247	0.08	68.5	78	100	5.6	2	3	★★★	2	healthy
Flavoured yoghurt	09C10095	120	240	401	2	11.6	60	20	0.1	4.7	2	★★★	2	healthy
Peanuts (roasted, salted)	11B10201	30	60	2667	9.18	4.4	335	100	6.2	25.1	−6	★★★★	4	less healthy
Muesli bar	12C10415	25	50	1713	3.42	23.6	128	20	7.7	6.3	6	★★★	9	less healthy
Muffin	02E10477	90	180	1516	1.84	25.9	343	10	1.4	4.8	12	★★	12	less healthy
Potato chips	10D10155	25	50	2233	6.8	2.2	415	0	3.5	4.7	13	★★	12	less healthy
Nut bar	12C10531	30	60	2248	10.57	20.7	21	70	8.4	15.7	9	★★✧	13	less healthy
Protein bar	02C20379	30	60	1811	6.97	9	203	0	1.4	34.2	13	★★	13	less healthy
Ice cream	09D10212	40	80	788	7.16	18.4	48	0	0	3.7	13	★★	13	less healthy
Lollies	12C10423	50	100	1350	0	50.6	110	0	0	5.3	16	★✧	15	less healthy
Carrot cake	02E10440	100	200	1582	4.02	30	336	10	1.5	4.3	16	★✧	15	less healthy
Popcorn	10D10135	20	40	2115	12.6	0.6	645	0	8.5	9.1	16	★✧	18	less healthy
Chocolate biscuits	02C20284	25	50	1841	8.38	23.9	308	0	1.9	6.2	20	★✧	19	less healthy
Chocolate	12C10407	20	40	2206	18.76	54.6	68	0	2.3	7.6	31	✧	23	less healthy

**Notes:** Ofcom NP: Ofcom Nutrient profile score. HSR: Health Star Rating Score. ★ equals 1 health star; ✧ equals 0.5 health stars.
